# Evaluation of Cowpea Landraces under a Mediterranean Climate

**DOI:** 10.3390/plants12101947

**Published:** 2023-05-10

**Authors:** Efstathia Lazaridi, Penelope J. Bebeli

**Affiliations:** Laboratory of Plant Breeding and Biometry, Department of Crop Science, Agricultural University of Athens, Iera Odos 75, 11855 Athens, Greece; e.lazaridi@aua.gr

**Keywords:** genotype × year interaction, local populations, seed yield, variability

## Abstract

Cowpea (*Vigna unguiculata* (L.) Walp.) yield is strongly influenced by environmental conditions. Average seed yield can decrease to a great extent when drought conditions occur, especially when they prevail during flowering and seed filling periods. Identifying genotypes presenting yield stability is one of the most important breeding goals. Local varieties or crop landraces are genetic resources that, despite exhibiting intermediate yield production capacity, present high yield stability in low-input cropping systems. The objective of this study was therefore to evaluate five selected cowpea landraces originated from different Greek islands under Mediterranean climatic conditions. A complete randomized block design with four replications was used during three consecutive cropping seasons. Many phenological and agronomic traits studied showed statistically significant genotype × experimental year interaction, while there was a strong experimental year effect. Among the landraces studied, local population VG23 from Kythira Island was the most productive under the experimental climatic and soil conditions, while local population VG2 from Lemnos Island was characterized by low seed productivity. Conclusively, our study showed that VG23 landrace is a promising genetic material to be used for seed yield improvement.

## 1. Introduction

Cowpea (*Vigna unguiculata* (L.) Walp.) seed and fresh pod yield is usually strongly influenced by prevailing environmental conditions, expressing significant environmental effects (E) and genotype × environment interactions (G × E) [1,2,3,4,5,6,7]. Average seed yield can especially be decreased when drought conditions occur during the flowering and seed filling periods [8,9,10]. Identification of genotypes expressing yield stability under different environments and throughout cropping seasons is considered one of the most important breeding goals [5,11], while yield is also recorded as the most desirable trait for farmers [12,13,14]. Many cowpea genotypes have been evaluated under diverse experimental environments for their seed yield, leading to the identification of promising breeding or genetic material [2,15,16,17,18,19,20]. However, yield is a complex and difficult trait to be directly improved, as it exhibits low heritability and pleiotropic effects [21]. Broad-sense heritability (*H*) for seed yield of cowpea ranges from 38.36% to 90.91% [22,23,24,25,26], while the genetic advance recorded in previous studies fluctuates from 4.55 to 8.25 [23,25].

Several plant morphological traits have been found to be positively associated with cowpea seed yield, such as plant height [27,28], number of inflorescences per plant [29,30,31], number of branches [29,32], pod length [2,27,30,33,34], number of pods per plant [2,27,28,31,33,34,35,36], number of seeds per plant [28], number of seeds per pod [2,27,29,30,31,33,36], pod weight [36], and hundred-seed weight [30,35]. In contrast, plant phenological traits’ associations with seed yield present inconsistency among studies, as days from sowing to flowering stage and to plant maturity have been either negatively [30,31,32,37] or positively related to seed yield [25,35]. Moreover, Gerrano et al. [38] did not observe any of the ten agronomic and morphological traits they studied, such as plant length, pod length, and number of pods per plant, to be significantly associated with seed yield (t ha^−1^) while evaluating twenty selected genotypes.

Currently, we are at a point at which the observed extreme climatic conditions may affect the stability of various crops yield globally [39,40,41]. The ability of a crop to produce consistent yields in different environments and under changing weather conditions therefore comes to the fore. Utilization of crop wild relatives (CWRs) in breeding could improve crop adaptability under adverse climate conditions. However, difficulties are faced regarding the introgression of genes from CWR into cultivated cowpea types due to incompatibility barriers [42]. On the other hand, landraces consist of genetic material with intermediate yield, production efficiency, and yield stability when they are cultivated in low-input cropping systems [43,44] and are easier to access and use in breeding for desirable traits compared to crop wild relatives. Therefore, they are important in terms of stable yield production [45,46] and of improving resistance to various abiotic stresses [47,48,49].

Cowpea cultivation in Southern Europe, including Mediterranean countries, starts in late spring (late April) and lasts until the beginning of autumn (early October) [1,50,51]. During the summer period, cowpea is confronted with water scarcity and high air temperatures, like in many other areas of the world [52,53], while in many cases, cultivation faces additional limiting soil factors [54]. In the countries around the Mediterranean basin, a remarkable number of cowpea landraces are still cultivated on a small scale by farmers mainly for their own use and consumption of either their young, tender pods or their seeds, which are rich in protein, carbohydrates, and nutrients [55,56]. These landraces could serve as important sources of adaptive traits and resistance to drought for the upcoming climatic changes [57,58,59,60].

The evaluation of cowpea landrace material originated from Southern European countries is considered limited in proportion to the number of local varieties that are available. The aim of this study was therefore to evaluate five selected cowpea landraces of Greek origin, with interesting morphological traits adapted to different microclimates with regards to their phenological characteristics and traits related to seed yield.

## 2. Results

### 2.1. Plant Phenological and Agronomical Traits

There was a statistically significant interaction among the accessions and the experimental years for all the phenological traits studied, with the exception of days from sowing till the appearance of mature pods, in 50% of the plants (DMAT) (Table 1). The accessions differed statistically significantly from each other regarding all three phenological characteristics studied, while statistically significant differences were observed among the experimental years (*p* ≤ 0.001).

Days from sowing to 50% flowering (DFL) during the first experimental year (2015) ranged from 60.67 days (VG2) to 68.67 days (VG3), during 2016 ranged from 60.17 days (VG23) to 75.17 days (VG3), and in the third experimental year (2017), they ranged from 63.67 days (VG23) to 80 days (IT97K-499-35). Overall, VG2 (Atsiki, Lemnos), VG4 (Marathi, Mykonos), and VG23 (Logothetianika, Kythira) showed earlier flowering than the rest of the accessions (Table 1). VG2 (Atsiki, Lemnos) required fewer days from sowing to 50% pod ripening (DMAT) than the other accessions, while VG20 (Mitilinioi, Samos) was the latest maturated one. Flowering duration (FDUR) lasted in 2015 from 58 (VG2) to 95.67 days (IT97K-499-35), in 2016 from 25.75 days (VG4) to 74.33 days (IT97K-499-35), and in 2017 from 81.33 days (VG2, VG3) to 89.66 days (IT97K-499-35) (Table 1). Of the three experimental years, flowering duration was statistically significantly reduced in the second experimental year (2016) (Table 1).

There was not a statistically significant interaction of accession x experimental year for the number of pods per plant, pod length, seed weight per plant, and number of seeds per plant (Table 2). Seed weight per plant did not differ statistically significantly among the three experimental years and the accessions, while pod length and hundred-seed weight did not differ statistically significantly among the experimental years (Table 2).

**Figure 1:**
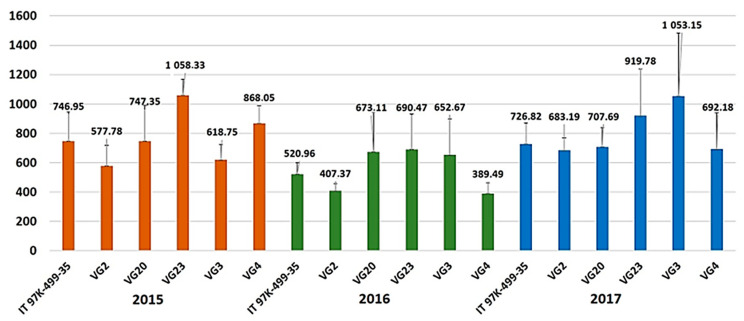
Seed yield (kg ha^−1^) for each accession and each experimental year. Seed yield for each accession for the three consecutive experimental years (2015, 2016, 2017) is presented in orange, green, and blue, respectively.

Plant height (PH) ranged from 25.08 cm (VG2) to 70.94 cm (VG23) in 2015, from 19.82 cm (VG2) to 41.44 cm (VG23) in 2016, and from 25.49 cm (IT97K-499-35) to 35.61 cm (VG3) in 2017 (Table 2). Average number of seeds per pod (SPOD) ranged from 5.27 (VG3) to 9.82 (VG23) in 2015, from 5.05 (VG4) to 14.39 (VG3) in 2016, and from 5.46 (VG4) to 7.79 (VG23) in 2017. Hundred-seed weight (100 SW) ranged from 14.05 g (VG2) to 22.49 g (VG4) in 2015, from 13.19 g (VG2) to 24.79 g (VG4) in 2016, and from 14.14 g (VG2) to 22.56 g (VG4) in 2017 (Table 2). Pod length (PODL) differed statistically significantly only among the accessions, with local population VG4 (Marathi, Mykonos) presenting the shortest mean pod length (9.55 cm) (Table 2). Number of pods per plant (NPOD) and number of seeds per plant (NSEED) differed only among the experimental years; during the second experimental year, these two traits compared to the other two experimental years showed lower averages (Table 2). Seed yield (kg ha^−1^) varied between 577.78 kg ha^−1^ (VG2-Atsiki, Lemnos) and 1058.33 kg ha^−1^ (VG23-Logothetianika, Kythira) in 2015, between 389.49 kg ha^−1^ (VG4-Marathi, Mykonos) and 690.47 kg ha^−1^ (VG23-Logothetianika, Kythira) in 2016, and between 683.19 kg ha^−1^ (VG2-Atsiki, Lemnos) and 1053.15 kg ha^−1^ (VG3-Alinda, Leros) in 2017 (Figure 1). However, seed yield (kg ha^−1^) did not differ statistically significantly among the accessions and the experimental years, while there was not a statistically significant interaction between accessions and experimental years.

Coefficients of variation (CV%) varied among experimental years for each trait under study (Table 3). Among the traits, days from sowing to 50% of flowering (9.47%) and days from sowing to 50% of pod ripening (9.54%) showed the lowest CV, while the CV for seed number per plant (68.75%) and seed yield (kg ha^−1^) (50.94%) were quite high (Table 3).

Among the accessions, a particularly high coefficient of variation (44.58%) was presented by the local population VG4 (Marathi, Mykonos) regarding flowering duration, by the VG23 (Logothetianika, Kythira) landrace (40.88%) regarding plant height, and by the local population VG3 (Alinda, Leros), which presented high CV for number of seeds per pod (52.50%), seed weight per plant (62.68%), number of seeds per plant (86.24%) and seed yield (kg ha^−1^) (62.70%) (Table 4).

### 2.2. Correlations among Studied Traits

Statistically very strong positive correlations were shown between number of pods and seed weight per plant (r = 0.830, *p* ≤ 0.001), number of pods and number of seeds per plant (r = 0.880, *p* ≤ 0.001), and number of pods and seed yield (kg ha^−1^) (r = 0.830, *p* ≤ 0.001) (Table 5). Strong positive correlations were observed between pod length and number of seeds per pod (r = 0.768, *p* ≤ 0.001) and between seed weight per plant and number of seeds per plant (r = 0.774, *p* ≤ 0.001). Pod length and number of seeds per plant were also positively correlated with seed yield (kg ha^−1^) with r = 0.534, *p* ≤ 0.001 and r = 0.774, *p* ≤ 0.001, respectively (Table 5).

### 2.3. Principal Component Analysis

Principal component analysis (PCA) was performed to reduce the dimensionality of the data, to study the contribution of each trait to the variability observed, as well as to illustrate the highest yield accessions. PCA showed that 79.88% of the total variation can be explained through the first three principal axes. Plant height, number of pods per plant, seed weight per plant, number of seeds per plant, and seed yield (kg ha^−1^) were related to the first principal component (PC1, 41.58%). Pod length and number of seeds per pod were related to the second principal component (PC2, 22.77%), while days from sowing to 50% of flowering, days from sowing to 50% of pods maturity, and flowering duration were related to the third principal component (PC3, 15.53%) (Table 6).

**Figure 2:**
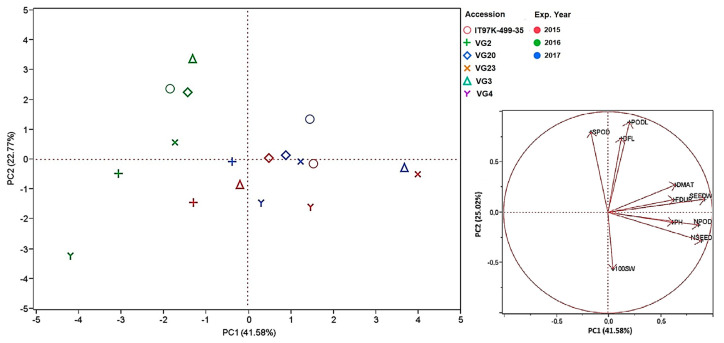
Classification of accessions through principal component analysis (PCA) and diagrammatic representation of the eigenvectors of studied traits. DFL: days from sowing to 50% flowering, FDUR: flowering duration, DMAT: days from sowing to 50% pod maturity, PH: plant height, NPOD: number of pods per plant, PODL: pod length, SPOD: number of seeds per pod, SEEDW: seed weight, NSEED: number of seeds per plant, 100 SW: hundred-seed weight. Accessions are presented with different symbols, while experimental years are indicated by different colors (2015: red, 2016: green, and 2017: blue).

During the second experimental year, accessions presented shorter flowering duration, earlier pod maturity, lower number of pods per plant, lower number of seeds per plant, and lower seed yield (kg ha^−1^) than in the other two experimental years. Therefore, the accessions in the second experimental year (presented in green color) were grouped separately (second and third quadrant) from the two other experimental years through principal component analysis (PCA) (Figure 2).

Accessions in the experimental years 2015 and 2017 were grouped in the first, third, and fourth quadrants, with the highest yield accessions to be depicted in the first and fourth quadrants. Landraces VG3 and VG23 presented as the highest yield accessions during 2017 and 2015, respectively, while VG4 was the lowest yield accession in 2016 (Figure 2). Most accessions expressed similar values for every studied trait for each accession under the 2015 and 2017 experimental years and therefore were depicted at close distances, with the exception of local population VG3 (Alinda, Leros) and local population VG23 (Logothetianika, Kythira). Landrace VG2 (Atsiki, Lemnos) was among the least productive accessions in all three experimental years (Figure 2).

## 3. Discussion

The investigation and promotion of landrace cultivation have been increased in recent years due to the extreme weather changes observed [48] and the high adaptability that landraces often present. Landraces, due to the remarkable variability that they usually possess, represent important sources of tolerance to abiotic stresses [61] while at the same time enhance sustainable cropping systems [62]. Although cowpea is a summer cultivated species in Europe and is considered as a resistant plant species to drought [63], it is very sensitive to water scarcity and high air temperature prevalence during the stages of flowering, fruiting, and pod-filling [8,9,10].

The accessions evaluated were practically classified into two groups based on the days needed from sowing to achieve 50% of flowering. The first group contained the early flowering landraces named VG2 (Atsiki, Lemnos), VG4 (Marathi, Mykonos), and VG23 (Logothetianika, Kythira), while the second included the late flowering accessions named VG3 (Alinda, Leros) and VG20 (Mitilinioi, Samos) and the improved line IT97K-499-35. Cowpea genotypes with short biological cycles and short flowering times are able to avoid the water scarcity that often prevails in the area during the summer months [64], while genotypes with long biological cycles seem to cope better under high temperatures as they gradually enter flowering and podding stages [65,66]. Mixtures of early- and late-flowering genotypes could also be used, aiming to deal with the negative effect of combined drought and high temperature on seed production [67,68,69]. Therefore, cowpea landraces evaluated in our study are diverse and represent promising material for high-air-temperature and drought tolerance or avoidance. In particular, the local population VG2 from Atsiki, Lemnos which was characterized by early flowering, a relatively low coefficient of variation of flowering duration (CV = 22.54%), and a determinate growth habit could be suitable material for drought avoidance [70].

Coefficients of variation calculated in the present study for days from sowing to 50% flowering and fruit setting were CV = 9.47% and CV = 9.54%, respectively, and are considered relatively low. Comparatively, high coefficients of variation for Greek cowpea landraces have been previously reported by Perrino et al. [71] for traits such as flowering initiation (22.1%) and pod length (23%), while the eight landraces that they investigated were characterized by earlier flowering than the accessions included in our study. Our collection also showed greater or equal coefficients of variation for number of pods per plant, number of seeds per pod, plant height, pod length, and number of seeds per plant compared to collections consisting of improved lines [72,73]. Landraces therefore presented non-uniformity in terms of these traits in comparison to improved lines which are usually characterized by high uniformity [74]. This is an agreement with the definition of landraces that they mostly comprise heterogeneous genetic material [15].

Cowpea landraces from Southern European countries have also presented high CV for traits that are related to seed yield [59]. Lower CV was recorded overall in our study for plant height (38.36%), number of pods per plant (44.01%), number of seeds per plant (68.75%), and hundred-seed weight (18.65%) in comparison to the total CV recorded by Carvalho et al. [59]. Among the accessions, local population VG23 (Logothetianika, Kythira) showed great variation among the experimental years for its plant height. This fact is reasonable as this landrace has been previously characterized by high diversity regarding its growth habit [56], a trait that affects plant height and the differences observed might therefore be due to random sampling of seeds at sowing.

The average cowpea seed yield in Greece fluctuates from 1 to 3.5 t ha^−1^ [16]. The average seed yield achieved during our study ranged from 0.39–1.05 t ha^−1^ and was therefore lower than the average seed yield reported for Greek conditions. The observed seed yield values were also lower than the average seed yield of cowpea landraces reported in other countries, such as in Ethiopia (2.05 t ha^−1^) and Brazil (1.05 t ha^−1^) [75,76]. Statistically significant differences have been recorded for cowpea landraces originated from Southern European countries regarding their seed yield, which ranged from 0.66 g m^−2^ to 3.12 g m^−2^ among three experimental locations and two experimental years [1]. The average seed yield previously recorded for twenty-two cowpea landraces, including one variety and the breeding line IT97K-499-35, was also greater (107.76 g m^−2^) than that observed in our study [59]. The low yield production recorded in our experiment could be due to various abiotic factors’ effects [77].

All landraces under study did not differ statistically significantly from each other, nor compared to the improved line, regarding their seed yield (kg ha^−1^). This fact is probably due to the unfavorable field conditions, which led to the production of low seed yield and did not allow landraces’ potential to be unfolded in our study [78,79,80]. In particular, the seed yield of the VG2 landrace from Atsiki of Lemnos was low (0.56 t ha^−1^ on average). This yield coincides with the threshold of its average productivity in its natural environment, Lemnos, in which seed yield ranges from 500 to 1500 kg per hectare (0.5–1.5 t ha^−1^), with an average yield of 700 kg per hectare (0.7 t ha^−1^). However, in Lemnos Island, cowpea cultivation is conducted without irrigation [81].

In particular, the low recorded yield could be based on the high presence of calcium carbonate (CaCO_3_ = 34.6%) and high pH (7.87) that characterized the specific experimental field, as both are considered as main limiting factors for the development of cowpea plants [82]. Seed yield of accessions derived in the present experiment are similar to those (0.81 t ha^−1^ on average) produced under similar adverse soil conditions [82]. According to Goenaga et al. [82], in order for a cowpea genotype to be considered tolerant to high soil alkalinity, it must be capable of producing a seed yield greater than or equal to 1 t ha^−1^. In the present study, local populations tested produced seed yield below this limit and therefore cannot be considered resistant to high-alkalinity conditions.

VG2 was among the least productive accessions during all the three experimental years, while many of its plants presented extensive chlorosis during their vegetative development. The chlorotic symptoms were probably due to the adverse soil conditions, as in Lemnos Island, cowpea cultivation does not take place in alkaline soils [81]. Furthermore, cowpea coexists mainly with nitrogen-fixing bacteria of the genus *Bradyrhizobium*, which form slow-growing populations [83,84]. Rhizobia strains isolated from the area of Atsiki, Lemnos formed a distinct group differentiated from other isolations that were taken in various locations of Greece [83]. This fact shows a possible specialization and symbiosis of these specific rhizobial strains and the local population from Lemnos Island (VG2). The extensive chlorosis in plants of the local population VG2 from Lemnos was overcome in most cases during their introduction to the reproductive stage, which indicates that chlorosis could be due to an incomplete symbiosis with the existing nitrogen-fixing bacteria. Cultivation of VG2 landrace in other soil environments could be therefore difficult and could create implications for attempting its utilization in a breeding program.

Seed production was reduced in the second experimental year for all tested accessions, with the exception of VG3 (Alinda, Leros Island) (Figure 1). Lower seed production during the second cultivation year could be due to the higher minimum temperature recorded in comparison to the other two years, which has been reported to lead to increased flower abortion and yield losses [85]. Increased flower abortion and lower yield production were also observed in four out of the five evaluated cowpea cultivars while applying the higher of two temperature regimes (20–26–33 °C and 24.8–30.8–37.8 °C) [86].

Despite that, there were not statistically significant differences among the accessions regarding seed yield, VG23 (Logothetianika, Kythira Island) was a landrace that exceeded the seed yield production of IT97K-499-35 breeding line in each one of the three experimental years. VG23 also presented a high number of pods and seeds per plant as well as large pod length, which are traits that have been strongly and positively related to seed yield (Table 5). Therefore, VG23 was considered to be the most productive accession in the current soil and climatic conditions. The statistically significantly higher genetic diversity that has been previously recorded for the VG23 landrace [56] in comparison to twenty-two other cowpea landraces of a Greek origin could be the reason for its increased seed yield production efficiency during the three experimental years. On the other hand, local population VG2 (Atsiki, Lemnos Island), which has been previously found to be one of the most homogeneous landraces among twenty-three local populations studied with Greek origin [56], was characterized by lower productivity but also by higher stability in comparison to the other landraces regarding many of the traits studied (Table 4). However, VG2 landrace stability could be only due to the impact of the unfavorable soil conditions of the experimental location used. Landraces unfold their potential when they are cultivated per se in the regions where they have been adapted, and despite their low productivity in ex situ cultivation, they should be further evaluated on-farm in each one’s region of origin. Therefore, the performance, including both productivity and stability of the examined landraces of the present experiment, should also be assessed in their region of origin as well as in other soil and climatic environments [49,87,88,89,90], as different cowpea landraces were found to be better adapted to diverse environments [59].

## 4. Materials and Methods

### 4.1. Plant Material and Experimental Design

**Figure 3:**
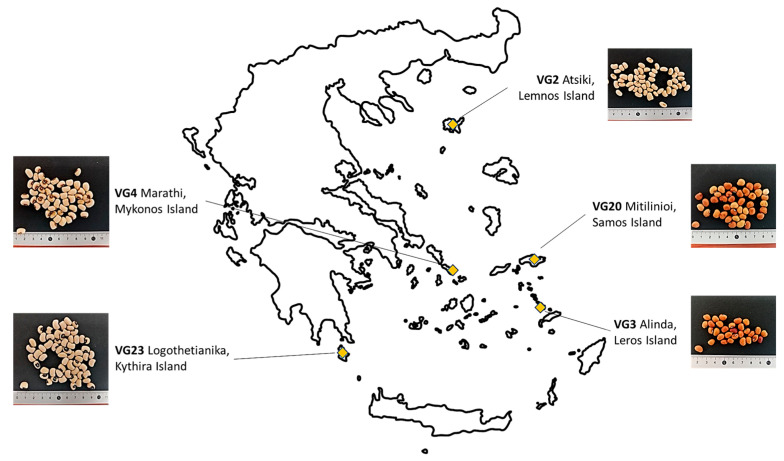
Accession codes, collection sites, and islands of cowpea landraces evaluated.

The experiment was conducted in a field at Agricultural University of Athens (AUA) (37°59′10″ Ν, 23°42′29″ Ε, altitude 24 m), during the spring–summer cultivation period for three consecutive years: 2015, 2016, and 2017. Sowing took place on 22 May 2015, on 15 May 2016, and on 29 May 2017. Five cowpea (*Vigna unguiculata* (L.) Walp.) landraces, named VG2 (Atsiki, Lemnos Island), VG3 (Alinda, Leros Island), VG4 (Marathi, Mykonos Island), VG20 (Mitilinioi, Samos Island), and VG23 (Logothetianika, Kythira Island), with origin from the Greek islands were evaluated (Figure 3).

Cowpea landraces were selected based on their interesting seed and pod morphological traits that were recorded through extensive characterization procedures [56]. The breeding line IT97K-499-35 originating from Nigeria was also used. A randomized complete block design (RCBD) with four replications was used. Each plot consisted of forty plants. Plant spacing between rows was 50 cm and spacing was 40 cm within each row.

### 4.2. Growth Conditions

**Figure 4:**
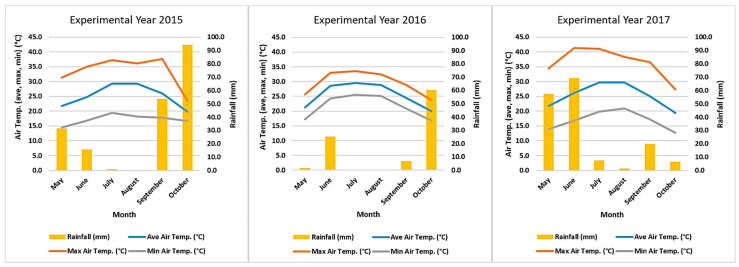
Mean, max, and min monthly air temperature (°C) and rainfall (mm) during the three consecutive experimental years (2015, 2016, and 2017).

The soil was sandy loam (SL) with a pH of 7.87 and a CaCO_3_ content of 34.6% at the depth of 25 cm (Supplementary Material S1). Plants were drip-irrigated and supplied with 1000 kg ha^–1^ of mineral fertilizer (NPK 11-15-15) as a base dressing. During the growing season, aphids were controlled by chemical means (Deltamethrin 2.5% *w*/*v*). Weeds were manually controlled. Monthly meteorological data throughout the three cultivation periods are presented in Figure 4.

### 4.3. Phenological and Yield Related Traits

Measurements of phenological and yield related traits were taken for ten central plants per plot. Phenological traits were recorded regarding days from sowing until 50% of the plants flowered (DFL), days from sowing to 50% pod maturity (DMAT), and flowering duration (FDUR), which was defined as the interval in days from the day of observation of the first open flower per plant to the observation of the last open flower per plant, including also the second flower flush that was recorded in some plants. Measurements related to yield included plant height (PH) (cm), number of pods per plant (NPOD), pod length (PODL) (cm), number of seeds per pod (SPOD), seed weight per plant (SEEDW) (g), number of seeds per plant (NSEED), and hundred-seed weight (100 SW) (g). Seed yield (SY) (kg ha^−1^) was then extrapolated from the total seed weight per plant.

### 4.4. Statistical Analysis

Residuals of all studied traits were subjected to normality tests and were checked for their homoscedasticity. Analysis of variance (ANOVA) was therefore applied, followed by Tukey’s (HSD) (*p* ≤ 0.05) means comparison method using the statistical software Statgraphics Centurion XVII. Coefficients of variation (CV%) were also calculated for each trait per accession and for each trait per experimental year for all accessions. Correlations between traits (Pearson correlation coefficients) were also investigated using the statistical package STATISTICA 8.0. Finally, a principal component analysis (PCA) was performed, aiming to study the contribution of each trait to the variability observed as well as to illustrate the highest yield accessions during the three experimental years by using the SAS statistical program JMP-8.

## 5. Conclusions

Promising variability was observed among the five landraces studied regarding their phenological and seed-yield-related traits. However, there were not statistically significant differences in terms of their seed yield (kg ha^−1^), a fact which is probably due to the unfavorable soil and climatic conditions that prevailed affecting the accessions’ performance and preventing them from unfolding their potential. Among the landraces, VG23 (Logothetianika, Kythira Island) was the most productive under the present soil and climate conditions, while the local population VG2 (Atsiki, Lemnos Island) was characterized by low seed productivity. Further evaluation in different environmental conditions is considered necessary, while VG23 could be utilized in a breeding program aiming to increase seed yield production.

## Figures and Tables

**Table 1:** Days from sowing until 50% of the plants flowered (DFL), days from sowing to 50% pod maturity (DMAT), and flowering duration (FDUR) for each accession and experimental year. Means ± SE in columns with different letters are statistically significantly different according to Tukey’s HSD means comparison method.

Experimental Year	Accession	DFL	DMAT	FDUR
2015	IT97K-499-35	68.00 ± 2.00 b–f	83.66 ± 1.33	95.67 ± 0.88 a
	VG2	60.67 ± 2.60 f	80.33 ± 3.18	58.00 ± 1.73 ef
	VG3	68.67 ± 0.88 b–f	94.00 ± 2.08	64.67 ± 0.66 d
	VG4	62.33 ± 1.76 de	85.00 ± 2.08	61.67 ± 2.40 de
	VG20	67.33 ± 2.33 b–f	96.67 ± 2.40	63.00 ± 4.51 e
	VG23	62.33 ± 1.20 de	91.67 ± 9.17	58.67 ± 1.20 ef
2016	IT97K-499-35	72.67 ± 1.45 a–d	87.00 ± 4.04	74.33 ± 0.33 cd
	VG2	64.58 ± 1.53 c–f	76.25 ± 2.25	50.00 ± 1.00 fg
	VG3	75.17 ± 1.59 ab	85.42 ± 1.42	41.67 ± 2.85 gh
	VG4	60.50 ± 0.76 f	76.25 ± 3.78	25.75 ± 2.27 i
	VG20	75.08 ± 1.58 ab	86.33 ± 2.33	45.33 ± 1.45 gh
	VG23	60.17 ± 1.30 f	75.17 ± 2.46	38.00 ± 1.00 h
2017	IT97K-499-35	80.00 ± 1.73 a	98.33 ± 2.73	89.66 ± 3.28 ab
	VG2	65.33 ± 0.67 b–f	90.00 ± 2.00	81.33 ± 0.88 bc
	VG3	74.00 ± 2.31 abc	95.00 ± 2.52	81.33 ± 0.88 bc
	VG4	64.67 ± 3.28 c–f	90.33 ± 2.91	83.00 ± 2.65 bc
	VG20	71.33 ± 2.40 a–e	92.00 ± 1.15	84.00 ± 1.73 bc
	VG23	63.67 ± 2.19 def	84.33 ± 1.20	82.00 ± 2.00 bc
Main effects				
2015		64.88 ± 1.01 b	88.56 ± 2.05 a	66.94 ± 3.26 b
2016		68.03 ± 1.65 a	81.07 ± 1.60 b	45.85 ± 3.63 c
2017		69.84 ± 1.62 a	91.67 ± 1.30 a	83.83 ± 0.99 a
IT97K-499-35		73.56 ± 1.95 a	89.67 ± 2.66 ab	86.55 ± 3.33 a
VG2		63.53 ± 1.15 b	82.19 ± 2.40 b	63.11 ± 4.74 b
VG3		72.61 ± 1.31 a	91.47 ± 1.83 a	63.11 ± 6.05 b
VG4		62.50 ± 1.25 b	83.86 ± 2.54 ab	56.81 ± 8.44 c
VG20		71.25 ± 1.55 a	91.67 ± 1.81 a	64.11 ± 5.77 b
VG23		62.06 ± 0.96 b	83.72 ± 3.65 ab	59.56 ± 6.40 bc
Statistical Significance				
Experimental Year		***	***	***
Accession		***	**	***
Exp. Year × Accession		*	ns	***

ns: non-significant, *: significant at the 0.05 level, **: significant at the 0.01 level, ***: significant at the 0.001 level.

**Table 2:** Plant height (PH), number of pods per plant (NPOD), pod length (PODL), number of seeds per pod (SPOD), seed weight per plant (SEEDW), number of seeds per plant (NSEED), and hundred-seed weight (100 SW) for each accession and experimental year. Means ± SE in columns with different letters are significantly different according to Tukey’s HSD means comparison method.

Experimental Year	Accession	PH (cm)	NPOD	PODL (cm)	SPOD	SEEDW (g)	NSEED	100 SW (g)
2015	IT97K-499-35	34.39 ± 8.11 b–d	19.58 ± 6.72	12.20 ± 0.51	9.23 ± 1.63 a–c	14.94 ± 4.00	129.27 ± 48.64	15.96 ± 0.07 cd
	VG2	25.08 ± 3.41 b–d	14.78 ± 2.18	9.31 ± 0.70	5.61 ± 0.59 c	11.56 ± 2.78	88.17 ± 17.28	14.05 ± 0.42 de
	VG3	45.33 ± 6.85 b	12.31 ± 1.23	10.59 ± 0.14	5.27 ± 0.19 c	12.38 ± 2.11	63.06 ± 7.28	18.76 ± 0.94 b
	VG4	37.08 ± 2.80 b–d	16.75 ± 2.84	11.81 ± 0.17	8.14 ± 0.76 a–c	17.36 ± 2.38	143.58 ± 29.07	22.49 ± 0.55 a
	VG20	26.49 ± 0.99 b–d	13.50 ± 3.53	12.50 ± 0.55	6.70 ± 0.34 bc	14.95 ± 4.84	93.33 ± 29.60	15.97 ± 0.85 cd
	VG23	70.94 ± 3.40 a	18.56 ± 0.75	14.48 ± 0.17	9.82 ± 0.40 a–c	21.17 ± 2.18	171.67 ± 12.51	17.20 ± 0.36 bc
2016	IT97K-499-35	22.96 ± 3.22 cd	8.83 ± 1.52	15.20 ± 0.93	12.65 ± 0.32 ab	10.42 ± 1.60	34.84 ± 0.91	15.74 ± 0.26 c–e
	VG2	19.82 ± 0.93 d	12.81 ± 1.52	8.98 ± 3.31	8.07 ± 2.78 a–c	8.15 ± 1.00	52.10 ± 8.74	13.19 ± 0.72 e
	VG3	27.70 ± 5.68 b–d	12.81 ± 0.69	8.98 ± 3.31	14.39 ± 0.98 a	13.05 ± 4.93	28.21 ± 5.75	15.39 ± 0.19 c–e
	VG4	23.92 ± 0.74 cd	7.89 ± 0.93	5.33 ± 1.68	5.05 ± 1.65 c	7.79 ± 1.43	44.08 ± 8.52	24.79 ± 1.19 a
	VG20	29.13 ± 2.30 b–d	8.69 ± 3.05	15.97 ± 4.05	11.01 ± 1.03 a–c	13.46 ± 5.43	26.59 ± 4.79	16.68 ± 0.70 b–d
	VG23	41.44 ± 6.80 b–d	9.92 ± 2.89	15.73 ± 3.53	10.67 ± 1.52 a–c	13.81 ± 4.83	30.70 ± 471	17.14 ± 0.40 bc
2017	IT97K-499-35	25.49 ± 1.57 b–d	15.72 ± 2.21	13.71 ± 0.96	7.59 ± 0.46 bc	14.54 ± 2.89	108.28 ± 22.37	15.95 ± 0.17 c–e
	VG2	29.48 ± 3.58 b–d	10.97 ± 1.64	11.78 ± 0.19	5.87 ± 0.13 c	13.66 ± 1.72	59.47 ± 8.03	14.14 ± 0.16 de
	VG3	35.61 ± 1.21 b	21.32 ± 6.84	12.42 ± 1.61	6.56 ± 1.66 bc	21.06 ± 8.59	132.49 ± 51.09	19.03 ± 0.14 b
	VG4	35.12 ± 1.57 b–d	13.62 ± 4.00	11.52 ± 2.08	5.46 ± 1.82 c	13.84 ± 8.18	82.47 ± 46.27	22.56 ± 0.08 a
	VG20	32.60 ± 1.85 b–d	15.71 ± 2.85	12.13 ± 1.40	6.54 ± 1.03 bc	14.15 ± 2.61	97.54 ± 25.99	15.12 ± 0.18 c–e
	VG23	30.61 ± 3.58 b–d	13.66 ± 4.05	13.48 ± 1.08	7.79 ± 1.00 bc	18.40 ± 6.38	103.71 ± 40.96	15.76 ± 0.12 c–e
Main effects								
2015		39.89 ± 4.11a	15.91 ± 1.35 a	11.81 ± 0.42	7.46 ± 0.50 b	15.39 ± 1.35	114.85 ± 13.03 a	17.41 ± 0.68
2016		27.50 ± 2.17b	9.92 ± 0.85 b	13.25 ± 1.53	10.31 ± 0.91 a	11.11 ± 1.40	36.08 ± 3.07 b	17.16 ± 0.91
2017		31.49 ± 1.18b	15.17 ± 1.57 a	12.51 ± 0.50	6.63 ± 0.45 b	15.94 ± 2.09	97.33 ± 13.43 a	17.09 ± 0.70
IT97K-499-35		27.62 ± 3.09bc	14.71 ± 2.61	13.70 ± 0.60 a	9.82 ± 0.90 a	13.30 ± 1.66	90.80 ± 21.07	15.88 ± 0.10 c
VG2		24.79 ± 2.01c	12.85 ± 0.98	10.02 ± 1.07 a	6.51 ± 0.91 bc	11.12 ± 1.27	66.58 ± 8.18	13.80 ± 0.29 d
VG3		36.21 ± 3.64b	15.01 ± 2.67	13.76 ± 1.64 a	8.74 ± 1.53 a–c	15.50 ± 3.24	74.58 ± 21.44	17.73 ± 0.65 b
VG4		32.04 ± 2.26bc	12.75 ± 1.94	9.55 ± 1.31 b	6.22 ± 0.89 c	13.00 ± 2.86	102.02 ± 23.85	23.28 ± 0.53 a
VG20		29.41 ± 1.26bc	12.63 ± 1.89	13.53 ± 1.39 a	8.08 ± 0.85 a–c	14.19 ± 2.24	72.49 ± 16.22	15.92 ± 0.39 c
VG23		47.66 ± 6.50a	14.04 ± 1.92	14.56 ± 1.12 a	9.43 ± 0.69 ab	17.79 ± 2.62	90.04 ± 21.56	16.70 ± 0.28 bc
Statistical Significance								
Experimental Year		***	*	ns	***	ns	***	ns
Accession		***	ns	*	*	ns	ns	***
Exp. Year × Accession		***	ns	ns	*	ns	ns	**

ns: non-significant, *: significant at the 0.05 level, **: significant at the 0.01 level, ***: significant at the 0.001 level.

**Table 3:** Coefficients of variability (CV%) per experimental year and for the total of the three years for each trait for all the studied accessions.

Experimental Year	DFL	FDUR	DMAT	PH	NPOD	PODL	SPOD	SEEDW	NSEED	100 SW	SY
2015	6.60%	20.68%	9.84%	43.68%	36.03%	14.99%	28.57%	37.17%	48.12%	16.65%	37.18%
2016	10.26%	33.63%	8.38%	33.49%	36.52%	48.92%	37.61%	53.74%	36.06%	22.54%	53.75%
2017	9.85%	5.03%	6.01%	15.95%	43.85%	17.13%	28.95%	55.65%	58.53%	17.30%	55.65%
Total CV%	9.47%	30.11%	9.54%	38.36%	44.01%	32.24%	38.84%	50.93%	68.75%	18.65%	50.94%

DFL: days to 50% flowering from sowing, FDUR: flowering duration, DMAT: days to 50% pod maturity from sowing, PH: plant height, NPOD: number of pods per plant, PODL: pod length, SPOD: number of seeds per pod, SEEDW: seed weight per plant, NSEED: number of seeds per plant, 100 SW: hundred-seed weight, SY: seed yield.

**Table 4:** Coefficients of variation (CV%) for each trait and accession.

Accession	DFL	FDUR	DMAT	PH	NPOD	PODL	SPOD	SEEDW	NSEED	100 SW	SY
IT97K-499-35	7.96%	11.53%	8.89%	33.56%	53.33%	13.11%	27.37%	37.49%	69.61%	1.87%	37.50%
VG2	5.43%	22.54%	8.75%	24.36%	22.88%	32.10%	41.91%	34.31%	36.85%	6.26%	34.32%
VG3	5.42%	28.78%	6.02%	30.13%	53.43%	35.80%	52.50%	62.68%	86.24%	10.98%	62.70%
VG4	6.01%	44.58%	9.10%	21.16%	45.60%	41.13%	42.75%	65.99%	71.83%	6.90%	65.99%
VG20	6.52%	27.02%	5.93%	12.85%	44.81%	30.81%	31.55%	47.40%	67.14%	7.40%	47.40%
VG23	4.64%	32.22%	13.08%	40.88%	40.95%	22.99%	21.83%	44.23%	70.13%	5.12%	44.22%

DFL: days to 50% flowering from sowing, FDUR: flowering duration, DMAT: days to 50% pod maturity from sowing, PH: plant height, NPOD: number of pods per plant, PODL: pod length, SPOD: number of seeds per pod, SEEDW: seed weight per plant, NSEED: number of seeds per plant, 100 SW: hundred-seed weight, SY: seed yield.

**Table 5:** Correlations between the studied traits according to Pearson correlation coefficients.

Trait	DFL	DMAT	PH	NPOD	PODL	SPOD	SEEDW	NSEED	100 SW	SY
DFL	0.351	0.512	−0.262	0.075	0.349	0.299	0.126	−0.036	−0.251	0.126
FDUR		0.506	0.004	0.348	0.048	−0.202	0.209	0.386	−0.224	0.209
DMAT			0.191	0.205	0.228	−0.110	0.265	0.244	−0.024	0.265
PH				0.353	0.232	−0.002	0.377	0.421	0.158	0.377
NPOD					0.250	−0.001	0.830	0.880	−0.011	0.830
PODL						0.768	0.540	0.080	−0.282	0.534
SPOD							0.240	−0.080	−0.281	0.240
SEEDW								0.774	0.044	1.000
NSEED									0.103	0.774
100 SW										0.044

DFL: days to 50% flowering from sowing, FDUR: flowering duration, DMAT: days to 50% pod maturity from sowing, PH: plant height, NPOD: number of pods per plant, PODL: pod length, SPOD: number of seeds per pod, SEEDW: seed weight per plant, NSEED: number of seeds per plant, 100 SW: hundred-seed weight, SY: seed yield. Statistical significance at the levels of ≤0.05, ≤0.01, and ≤0.001.

**Table 6:** Variation explained by each axis, contribution of each trait under study to the total diversity. **Bold** shows the axis to which each trait mainly contributed.

Trait	PC1 (41.58%)	PC2 (22.77%)	PC3 (15.53%)
Days to 50% flowering	−0.117	0.433	**0.767**
Flowering duration	0.363	−0.207	**0.780**
Days to 50% pod maturation	0.415	−0.008	**0.728**
Plant height	**0.799**	0.125	−0.315
Number of pods per plant	**0.776**	−0.234	0.355
Pod length	0.226	**0.930**	0.194
Number of seeds per pod	−0.058	**0.933**	−0.100
Seed weight per plant	**0.939**	0.156	0.173
Number of seeds per plant	**0.864**	−0.324	0.190
Hundred-seed weight	0.181	−0.399	−0.430
Seed yield (kg ha^−1^)	**0.939**	0.156	0.173

## Data Availability

Not applicable.

## Supplementary Materials

The following supporting information can be downloaded at: https://www.mdpi.com/article/10.3390/plants12101947/s1, Supplementary Material S1. Soil analysis performed at a depth of 0–25 cm. All the necessary Supplementary Material (S1) mentioned in the text of the manuscript are attached separately with the manuscript.
