# Supplementary material for: Evaluation of Cowpea Landraces under a Mediterranean Climate
Source: Plants (Basel). 2023 May 10;12(10):1947. doi: 10.3390/plants12101947 (PMC10223609; doi:10.3390/plants12101947)
Supplement: Supplementary file 1 [file plants-12-01947-s001.zip › plants-2353642-supplementary.pdf]

Table S1. Soil analysis performed in a depth of 0-25 cm.

Physicochemical Parameter	Soil Analysis Values
Granulometric Analysis	69.20% sand
	12.60% silt
	18.20% clay
Soil Texture (Texture Class)	SL (Sandy Loam)
pH	7.87
Electric Conductivity (EC)	0.38 mS/cm
Calcium Carbonite	34.6%
Organic matter	3.82%
Total Nitrogen	0.22%
NO_3	18.95 ppm
NH_4	6.92 ppm
Assimilable P	32.22 ppm
Exchangeable Ca	7142.50 ppm
Exchangeable Mg	440.00 ppm
Exchangeable K	522.00 ppm
Exchangeable Na	196.00 ppm
